# Comprehensive Analysis of Aspergillus nidulans PKA Phosphorylome Identifies a Novel Mode of CreA Regulation

**DOI:** 10.1128/mBio.02825-18

**Published:** 2019-04-30

**Authors:** Liliane F. C. Ribeiro, Cynthia Chelius, Karthik R. Boppidi, Nisha S. Naik, Simin Hossain, Jessica J. J. Ramsey, Jyothi Kumar, Lucas F. Ribeiro, Marc Ostermeier, Bao Tran, Young Ah Goo, Leandro J. de Assis, Mevlut Ulas, Ozgur Bayram, Gustavo H. Goldman, Stephen Lincoln, Ranjan Srivastava, Steven D. Harris, Mark R. Marten

**Affiliations:** a Department of Chemical, Biochemical and Environmental Engineering, University of Maryland, Baltimore County (UMBC), Baltimore, Maryland, USA; b Center for Plant Science Innovation, University of Nebraska, Lincoln, Nebraska, USA; c Department of Chemical and Biomolecular Engineering, Johns Hopkins University, Baltimore, Maryland, USA; d Mass Spectrometry Center, University of Maryland School of Pharmacy, Baltimore, Maryland, USA; e Departamento de Ciências Farmacêuticas, Faculdade de Ciências Farmacêuticas de Ribeirão Preto, Universidade de São Paulo, São Paulo, Brazil; f Department of Biology, Maynooth University, Maynooth, County Kildare, Ireland; g Chemical & Biomolecular Engineering Department, University of Connecticut, Storrs, Connecticut, USA; h Faculdade de Ciências Farmacêuticas de Ribeirão Preto, Universidade de São Paulo, CEP 14040-903, São Paulo, Brazil; i Institute for Advanced Study, Technical University of Munich, Garching, Germany; University of Georgia

**Keywords:** CreA, phosphoproteomic, phosphosite, signal transduction, transcriptomic

## Abstract

In filamentous fungi, an important kinase responsible for adaptation to changes in available nutrients is cyclic AMP (cAMP)-dependent protein kinase (protein kinase A [PKA]). This kinase has been well characterized at a molecular level, but its systemic action and direct/indirect targets are generally not well understood in filamentous fungi. In this work, we used a *pkaA* deletion strain (Δ*pkaA*) to identify Aspergillus nidulans proteins for which phosphorylation is dependent (either directly or indirectly) on PKA. A combination of phosphoproteomic and transcriptomic analyses revealed both direct and indirect targets of PKA and provided a global perspective on its function. One of these targets was the transcription factor CreA, the main repressor responsible for carbon catabolite repression (CCR). In the Δ*pkaA* strain, we identified a previously unreported phosphosite in CreA, S319, which (based on motif analysis) appears to be a direct target of Stk22 kinase (AN5728). Upon replacement of CreA S319 with an alanine (i.e., phosphonull mutant), the dynamics of CreA import to the nucleus are affected. Collectively, this work provides a global overview of PKA function while also providing novel insight regarding significance of a specific PKA-mediated phosphorylation event.

## Abstract (precis)

The cyclic AMP (cAMP)-dependent protein kinase A (PKA) signaling pathway is well conserved across eukaryotes, and previous work has shown that it plays an important role in regulating development, growth, and virulence in a number of fungi. PKA is activated in response to extracellular nutrients and acts to regulate metabolism and growth. While a number of components in the PKA pathway have been defined in filamentous fungi, current understanding does not provide a global perspective on PKA function. Thus, this work is significant in that it comprehensively identifies proteins and functional pathways regulated by PKA in a model filamentous fungus. This information enhances our understanding of PKA action and may provide information on how to manipulate it for specific purposes.

## INTRODUCTION

Eukaryotic cells possess a multitude of signaling pathways that enable them to monitor their local environment and to rapidly respond in a precise manner when conditions change (1, 2). A prominent example is the cyclic AMP (cAMP)-dependent protein kinase A (PKA) pathway. In fungi, PKA signaling mediates a wide range of processes, including nutrient sensing (3), stress responses (4), regulation of metabolism (5), as well as development and pathogenicity (6, 7). Although the molecular basis for PKA action is well understood, with more than a hundred different crystal structures published (8), considerably less is known about its systemic action and impact on downstream effectors (9, 10). This is particularly true for filamentous fungi.

In Saccharomyces cerevisiae, PKA is largely responsible for glucose sensing and subsequent modulation of cellular responses (11). In Aspergillus fumigatus (12) and Neurospora crassa (13), PKA is activated in the presence of glucose, thereby inhibiting alternative carbon source usage due to carbon catabolite repression (CCR) (12, 14). CCR occurs when glucose is sensed, primarily via the Ras/cAMP pathway (15), leading to increased adenylate cyclase activity and higher levels of cAMP. Newly formed cAMP then binds to the regulatory subunit of PKA, releasing the catalytic subunits to start a protein phosphorylation cascade (12, 13). In yeast, once PKA is activated, it phosphorylates target substrates such as Maf1 (16), RCM-1 (17), Msn2 (18), Msn4 (18), Rim15 (19), Rap1 (20), pyruvate kinase (21), Sak1 (22), Sip1 (23), Adr1 (24), Bdp1 (25), and Yak1 (26). However, these targets are not sufficient to explain the broad action of PKA in different cellular phenotypes.

In Aspergillus nidulans, CCR is shown to be mediated mainly by the transcription factor CreA, a Cys2-His2 type DNA-binding zinc finger protein (27, 28). CreA is responsible for repressing the transcription of numerous genes encoding lignocellulolytic enzymes when glucose is present, thus preventing the utilization of alternative carbon sources (29). When the cell senses glucose, CreA is imported into the nucleus, binds to DNA, and represses transcription of several genes. However, the mechanisms that direct the localization of CreA, and its affinity for binding to DNA remain somewhat unclear in filamentous fungi (12). Previous work has shown that Trichoderma reesei CreA homologue Cre1 is phosphorylated by casein kinase II at Ser 241, which is essential for DNA binding (30). However, it has been suggested that Cre1 nuclear import is regulated by a different mechanism, perhaps via interactions with other proteins or protein turnover (30). In S. cerevisiae, nuclear localization of the CreA functional homologue Mig1p is regulated through phosphorylation by the kinase Snf1p (31). In the presence of glucose, Snf1p is inactivated, resulting in dephosphorylation of Mig1 and its retention in the nucleus (32). In A. nidulans, there is a lack of evidence confirming direct phosphorylation of CreA. However, some studies suggest that kinases in A. nidulans are responsible for CreA cellular localization because the deletion of *snfA* or *schA* prevents CreA from leaving the nucleus in derepressing conditions (33). While CreA contains sites that are predicted to be phosphorylated by PKA, their functional relevance has not yet been investigated (12).

Here we utilize a comprehensive phosphoproteomic screen to identify both direct and indirect targets of PKA. One of these targets is CreA, which is also found to be regulated at a transcriptional level. We identify a unique phosphorylation site on this protein which has not been previously described. Upon further functional investigation, we find that this site is required for the proper dynamics of CreA nuclear import during CCR.

## RESULTS AND DISCUSSION

To better understand the systemic action of PKA in A. nidulans, we carried out phosphoproteomic and transcriptomic differential analyses of two A. nidulans strains: a *pkaA* deletion strain (Δ*pkaA*) and its isogenic parent (34).

### Phosphoproteomic analysis.

To conduct phosphoproteomic analysis, we grew fungi in glucose to induce the PKA pathway. Our analysis resulted in the identification of 847 differentially phosphorylated peptides in the Δ*pkaA* strain (see Table S1 in the supplemental material). For comparison, Franck et al. (35) found 485 peptides differentially phosphorylated in Magnaporthe oryzae in a comparison between the wild type (WT) and a *cpkA* (PKA) deletion strain. Among the 847 peptides identified here, only 5 phosphopeptides (corresponding to 5 proteins) were found exclusively in the Δ*pkaA* strain. These five phosphopeptides included AN8281, AN10591, AN8035.2, and AN7474 (uncharacterized proteins) and AN1948 (*SPA10*), which is a septal pore-associated protein (36). In contrast, 842 identified phosphopeptides (from 554 proteins) were found exclusively in the control strain.

10.1128/mBio.02825-18.5 TABLE S1 Differentially phosphorylated peptides in the Δ*pkaA* strain. Download Table S1, XLSX file, 0.7 MB. Copyright © 2019 Ribeiro et al. 2019 Ribeiro et al. This content is distributed under the terms of the Creative Commons Attribution 4.0 International license.

**FIG 1:**
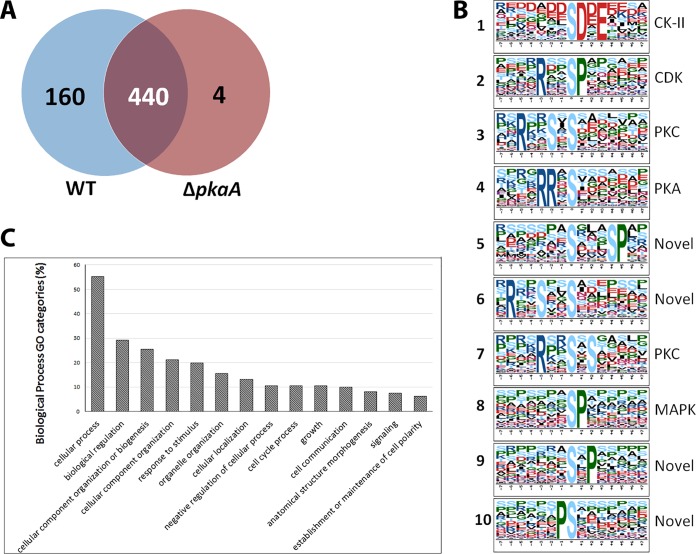
Phosphoproteome-wide effects on Aspergillus nidulans. (A) Venn diagram illustrating the distribution of phosphoproteins between the wild-type (WT) and Δ*pkaA* strains that were equally transcribed. (B) Ten most prevalent phosphorylation motifs (motif X) from differentially phosphorylated peptides. (C) Gene Ontology (GO) categories for proteins identified for biological function.

Because the presence or absence of PKA is expected to affect the transcription of a large number of genes, we used RNA-seq analysis to select for proteins within our phosphoproteomic data set that were differentially phosphorylated but transcribed at similar levels. This approach increases the probability that a differentially phosphorylated peptide was not an artifact of different protein expression levels. When only similarly transcribed genes (0.5 < Δ*pkaA*/WT < 2.0) are considered, we find 337 unique phosphosites from 160 proteins that are differentially phosphorylated (Fig. 1A).

Enriched motifs among the set of PKA targets identified by phosphoproteomics were identified using motif X (Fig. 1B). The 10 most prevalent motifs were compared to motifs identified in the literature: the acidic motif [SDxE] (motif 1) is recognized by casein kinase II (CK-II) (37), [RxxSP] (motif 2) is typically recognized by cyclin-dependent kinase (CDK) (38), [RxxSxS] (motifs 3 and 7) is a potential target of PKC (39), and [RRxS] (motif 4) is known to be recognized by PKA (37). [SP] (motif 8) is potentially recognized by a mitogen-activated protein kinase (MAPK) and ERK (40). We note that [SxxxSP] (motif 5), [RxxSxxS] (motif 6), [PS] (motif 9), and [SxP] (motif 10) appear to be novel as they have not been previously identified in the literature (37). We also note that the kinases predicted to phosphorylate the known motifs all play significant roles in the PKA pathway, acting as important mediators between active PKA and the cellular response to this activation.

All similarly transcribed and differentially phosphorylated proteins were categorized according to Gene Ontology (GO) for Biological Process (Fig. 1C). Consistent with previous findings, PKA impacts a broad range of proteins that are involved in a number of different biological processes. We find that PKA has a strong impact on a number of other kinases, as 28 were found to be differentially phosphorylated (Table S2). NetworKIN 3.0 was used to predict which kinase phosphorylates each phosphosite. Of these 28 kinases, 4 were predicted to be phosphorylated directly by PKA, including a hexokinase (AN7459), a Pom1 kinase homologue pomA (AN7678), the regulatory subunit of PKA (AN4987), and a choline kinase (AN0929). Of these, the first three (AN7459, AN7678, and AN4987) have phosphorylation motifs matching motif 4 in Fig. 1B. However, AN0929 was phosphorylated at a different motif (RTVS*).

10.1128/mBio.02825-18.6 TABLE S2 Differentially phosphorylated kinases in the Δ*pkaA* strain. Download Table S2, XLSX file, 0.01 MB. Copyright © 2019 Ribeiro et al. 2019 Ribeiro et al. This content is distributed under the terms of the Creative Commons Attribution 4.0 International license.

**FIG 2:**
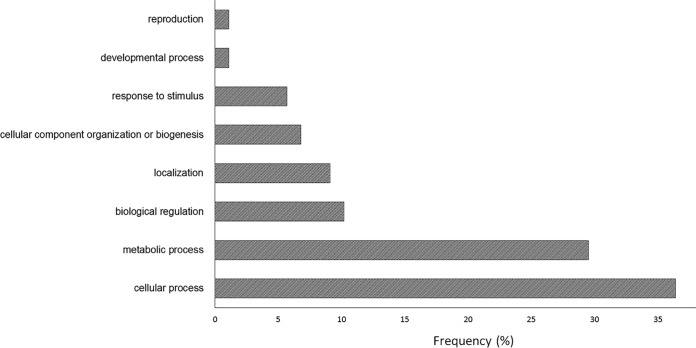
Biological function Gene Ontology (GO) categories for proteins predicted to be phosphorylated directly by PKA.

To identify additional direct PKA substrates, we searched for all differentially phosphorylated peptides that contained the classical PKA phosphorylation motif R/K-R/K-x-S and found 71 that matched it. GO categories for these phosphopeptides show that they are from proteins primarily related to cellular process (Fig. 2). Among these proteins, only one is a known PKA target, StuA. This implies that the remaining 70 are potentially new PKA substrates, none of which have been previously identified in the literature (Table S3). We assume the phosphopeptides with other motifs were phosphorylated indirectly, by another kinase, that itself may be a target of PKA.

10.1128/mBio.02825-18.7 TABLE S3 Potential direct targets of PKA. Download Table S3, XLSX file, 0.01 MB. Copyright © 2019 Ribeiro et al. 2019 Ribeiro et al. This content is distributed under the terms of the Creative Commons Attribution 4.0 International license.

One of the 70 proteins was a hexokinase, AN7459. A. nidulans contains two confirmed hexokinase-encoding genes (41, 42); AN7459 (*hxkA*), and AN2638 (*hxkB*). The AN4255 (*hxkC*) and AN2180 (*xprF*) genes encode atypical noncatalytic hexokinases that regulate protease production (40, 43). In our results, AN7459 was found to be equally transcribed and phosphorylated only in the WT, and AN4255 was differentially transcribed (three times less in the PKA^−^ strain). AN2638 and AN2180 were equally transcribed and not found to be phosphorylated in this work. de Assis et al. (29) reported a reduction in hexokinase activity in a *pkaA* knockout strain of A. nidulans compared to the WT, showing that PKA controls the activity of hexokinase. However, the mechanism by which hexokinase control occurs was not described. Therefore, according to the results obtained in this work, hexokinase activity could be controlled transcriptionally or by phosphorylation/dephosphorylation of HxkA or possibly HxkC.

Among the proteins that were equally transcribed, 19 transcription factors (TFs) were observed to be differentially phosphorylated (Table 1). One of the TFs identified as being phosphorylated in the presence of PKA is PacC. PacC is a zinc finger transcription factor, and in A. nidulans, it mediates regulation of both acid- and alkaline-expressed genes by ambient pH. In Cryptococcus neoformans, Rim101/PacC is responsible for adaptive response to changes in pH and has been shown to share downstream targets with PKA (44). Previous work and our data strongly suggest that PacC is an indirect target of PKA. Among the 19 transcription factors identified here, two (AreA [AN8667] amd AreB [AN6221]) are involved with regulation of nitrogen metabolism. Since PKA is known to affect different aspects of cellular growth and to be involved in the regulation of nitrogen metabolism (45), our results imply that these TFs are downstream effectors of the PKA pathway and further explain the broad action of PKA activation.

**TABLE 1:** Transcription factors equally transcribed and phosphorylated only in the WT

Phosphopeptide	Uniprot	Gene	Protein
TSSTPNTAQLLR	P17429	*areA* AN8667	Nitrogen regulatory protein AreA
SIDTQASRPPTMQPASQTSGDNTSTDSR	Q5AU19	AN8211.2 ANIA_08211	PHD transcription factor (Rum1)
AASESMDLSSDDKESGER	Q00202	*pacC* AN2855	pH response transcription factor PacC/RIM101
AESPEASTEAEPFEER	Q5BCK1	AN1729.2 ANIA_01729	PrnA protein
ALLDPTEIIQSPSSAKK	C8VQ48	ANIA_00162	Putative APSES transcription factor
KSGSDDDGSAGSGMVQEVK	Q5AVL9	AN7661.2 ANIA_07661	Putative bHLH transcription factor
RLDEPEDSVAETTTTTPPSQQPQEQTR	Q5BH27	AN0153.2 ANIA_00153	Putative Myb-like transcription factor
SMVADDDNRPTTQYNTSPTGTGSSR	C8VRL9	ANIA_01402	Putative Zn(II)2Cys6 transcription factor
SLSAGGYNATNSPTR	Q5B4H2	AN4558.2 ANIA_04558	Putative Zn(II)2Cys6 transcription factor
QQLASMSDAEIQK	Q5B9K9	AN2771.2 ANIA_02771	Transcription factor Rba50
LASPVSPSPAVK	Q5AYD4	AN6696.2 ANIA_06696	Transcription factor Tos4
MSDEWESEGEEDIAAPEEK	Q5BF86	AN0794.2 ANIA_00794	Transcription initiation factor TFIID, 31-kDa subunit
LQLADDGGEESDDEPIMSSR	Q5B3I6	AN4894.2 ANIA_04894	Transcriptional activator Spt7
SEAGTPPLGVSQGYR	G5EB05	AN6221.2 ANIA_06221	Uncharacterized protein
SSLASLNTTDSR	G5EB07	*nsdD* AN3152.2	Uncharacterized protein
KPSASILVPR	C8V8D1	ANIA_04502	BZIP transcription factor
SDSGEFPPIASK	Q5BFB4	AN0766.2 ANIA_00766	C6 finger domain protein
ASSTASPVVTLAQPVPK	C8V7V5	ANIA_04585	CCR4-NOT transcription complex, subunit 3
RKTLTETPVGGPVGGVPLGLQPMK	P36011	*stuA* AN5836	Cell pattern formation-associated protein StuA

To validate our phosphoproteomic data and provide more information regarding substrates that may be direct targets of PKA, we used anti-GFP antibodies to carry out an immunoprecipitation of PKA. In this experiment, the proteins directly interacting with PKA were coprecipitated and identified via MS/MS. Among a total of 217 proteins identified (Table S4), 32 proteins were common in both phosphoproteomic and immunoprecipitation experiments (Table S5). While coimmunoprecipitation is often used to study protein-protein interactions, it frequently presents a limitation with contaminant proteins being coprecipitated as well (46), but it does provide insight regarding potentially direct PKA substrates.

10.1128/mBio.02825-18.8 TABLE S4 Immunoprecipitation of GFP-tagged PKA and identification of proteins interacting with PKA by MS/MS. Download Table S4, XLSX file, 0.04 MB. Copyright © 2019 Ribeiro et al. 2019 Ribeiro et al. This content is distributed under the terms of the Creative Commons Attribution 4.0 International license.

10.1128/mBio.02825-18.9 TABLE S5 Proteins identified by phosphoproteomics and immunoprecipitation of PKA-GFP. Download Table S5, XLSX file, 0.01 MB. Copyright © 2019 Ribeiro et al. 2019 Ribeiro et al. This content is distributed under the terms of the Creative Commons Attribution 4.0 International license.

To investigate PKA targets in Candida albicans, Cao et al. mutated the two PKA catalytic subunits and used differential phosphoproteomic analysis to identify proteins in which phosphorylation was affected by the mutations (43). Identified proteins were subjected to *in silico* analysis to determine potentially direct substrates of PKA. To do this, they analyzed the phosphorylated motif (R/K-R/K-x-S/T) and found 148 proteins that are likely direct PKA substrates. Among the proteins listed as potential PKA targets, there were five orthologs also identified in this study. The proteins identified in common are a kinase (AN10515/PRK1), a protein related to biosynthesis of pyrimidine (AN0565/pyrABCN), an ATP-dependent RNA helicase subunit 2 (AN8722/SUB2), a protein repressed during the mating process (AN0446/PSP1), and a protein required for cell wall chitin distribution, morphology, and hyphal growth (AN0979/BNI4) (43).

### Transcriptomic analysis.

**FIG 3:**
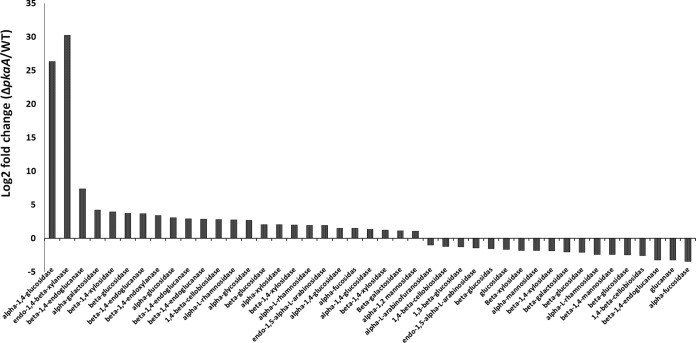
Hemicellulase transcription change (Δ*pkaA*/WT). Log_2_ fold change in the expression of glycosyl hydrolases when fungi were cultivated for 20 h in rich media with glucose as the carbon source.

We used an RNA-seq approach to assess differential gene expression in the *pkaA* deletion strain (Δ*pkaA*) and its isogenic parent. Because PKA is involved in many processes, deletion of *pkaA* affected gene expression very broadly. RNA-seq results showed that 125 TFs were differentially transcribed. A total of 44 TFs were downregulated and 81 were upregulated in the absence of PKA (Table S6). Of the TFs that were downregulated, CreA, an important carbon catabolite repressor in A. nidulans (14), had a 3.6-fold-lower expression level in the knockout strain. Since CreA represses transcription of cellulases and xylanases, we observed that the levels of expression of several genes related to carbohydrate metabolism change significantly [−1.0 < log_2_ (Δ*pkaA*/wt) < 1.0] in the PKA deletion strain compared to the WT (Fig. 3). Our data also imply that PKA regulation occurs by both differential phosphorylation of TFs and altered TF transcription levels.

10.1128/mBio.02825-18.10 TABLE S6 Differentially transcribed transcription factors in the Δ*pkaA* strain. Download Table S6, XLSX file, 0.03 MB. Copyright © 2019 Ribeiro et al. 2019 Ribeiro et al. This content is distributed under the terms of the Creative Commons Attribution 4.0 International license.

### CreA regulation.

**FIG 4:**
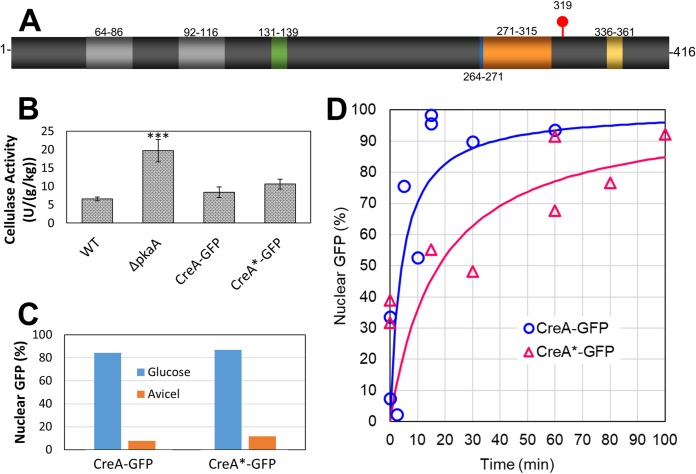
Comparison of wild type CreA-GFP and phosphonull strain containing CreA^S319A^ (CreA*-GPF). (A) CreA protein showing subdomains. The zinc fingers (light gray), polyalanine-rich region (green), acidic region (blue), conserved region (orange), and repressing region (yellow) subdomains are shown. (B) Comparison of cellulase activity expression during growth on Avicel as the substrate. The *** indicates significant difference between the Δ*pkaA* strain and all other strains. (C) Nuclear localization of CreA-GFP and CreA*-GFP when grown for 16 h in either glucose or Avicel. (D) Nuclear import of CreA-GFP and CreA*-GFP as a function of time after switch from ethanol to glucose as the carbon source. The lines are models fitted as described in the text.

Although our results showed numerous proteins that were equally transcribed and differentially phosphorylated, most of the proteins were uncharacterized or of unknown function. Therefore, we selected CreA, a transcription factor that has been well characterized in terms of the genes it regulates, the conditions in which it is active, and its functional localization, to study the effects of the phosphosites identified in our results. In the Δ*pkaA* strain, CreA was shown to be regulated both transcriptionally (i.e., 3.6-fold-lower expression in the Δ*pkaA* strain) and posttranslationally (S319 phosphorylated in the wild type and not in the Δ*pkaA* mutant). We note that in a previous A. nidulans study, we created a TAP-tagged version of CreA (47). We then grew this CreA:TAP strain in xylan, transferred it to glucose for various lengths of time (i.e., 5, 10, 15, and 30 min), and then immunoprecipitated CreA. We saw no physical interaction between CreA and the catalytic subunit of PKA (47). This evidence and the fact that we did not observe CreA when we immunoprecipitated PkaA:GFP (described above) strongly imply that CreA is not physically interacting with PkaA nor is it interacting in a very transient and/or weak manner. Further, a motif-based analysis (NetworKIN 3.0) predicts S319 to be a direct substrate of Stk22 kinase (AN5728). These data led us to hypothesize that PKA mediates CCR by at least two nonexclusive mechanisms. (i) It increases *creA* mRNA levels, thereby enhancing the repression of glycosyl hydrolases and other glucose-repressed genes. (ii) One kinase in the PKA pathway (possibly Stk22, AN5728), phosphorylates CreA to presumably enforce transcriptional repression of glycosyl hydrolases, among others. To test the hypothesis that phosphorylation of S319 plays a critical role in the ability of CreA to mediate CCR, we mutated CreA residue S319 (Fig. 4A), identified in our phosphoproteomic analysis, to an alanine (S319A) and fused this construct to GFP (CreA^S319A^::GFP). Subsequently, we investigated the expression of cellulase activity (Fig. 4B) and CreA::GFP nuclear localization (Fig. 4C and D; see also Fig. S1 in the supplemental material) in this strain.

10.1128/mBio.02825-18.1 FIG S1 Microscopy of CreA::GFP and CreA(S319A)::GFP for cellular localization. Download FIG S1, JPG file, 0.1 MB. Copyright © 2019 Ribeiro et al. 2019 Ribeiro et al. This content is distributed under the terms of the Creative Commons Attribution 4.0 International license.

In A. nidulans, CreA consists of two C2H2-type zinc fingers (necessary for DNA binding), an alanine-rich region, an acidic region, and a conserved region, followed by a region that is required for the repression mediated by CreA (Fig. 4A) (28, 48). Only the repressing region has been functionally characterized, and few studies have investigated CreA posttranslational regulation (28). As the main repressor in CCR, CreA inhibits the expression of numerous hydrolytic enzymes. In T. reesei, dephosphorylation of the CreA homologue Cre1 at residue S241 is necessary for binding to target DNA and repression of transcription (30). In another study, Alam et al. (49) have identified two peptides that were phosphorylated in CreA when A. nidulans was cultivated under repressing conditions and not phosphorylated under derepressing conditions. Among the two phosphopeptides, they found the sites S289, S312, and S319 to be phosphorylated only in the presence of glucose. To characterize the mutant CreA^S319A^ strain phenotype, we assayed for cellulase activity (Fig. 4B), xylanase activity (Fig. S2B), radial growth in carboxymethyl cellulose, allyl alcohol, and 2-deoxy-D-glucose (Fig. S2). In all cases, we find no phenotypic difference between the wild-type CreA and CreA^S319A^ strains. In accordance with a previous study (29), the *pkaA* deletion strain showed a significant increase in cellulase activity which was expected since transcription of several glucanases was higher in the deletion strain (Fig. 3). However, there was no difference in activity in the CreA^S319A^ strain (Fig. 4B). CreA is known to display nuclear localization in the presence of glucose (33), a repressive condition. However, upon derepression, CreA is dispersed throughout the cytoplasm (33). We found that localization of CreA^S319A^ was unaffected during growth in glucose (Fig. 4C). However, when the fungus was cultivated in derepressing conditions (i.e., ethanol) for 16 h and then switched to glucose, CreA^S319A^::GFP did not migrate to the nucleus at the same rate as the CreA::GFP in the control (WT) strain (Fig. 4D and Fig. S1). We used a simple, saturation, kinetic model to estimate saturation constants for both CreA::GFP and CreA^S319A^::GFP (equation 1).

(1) $$\text{Nuclear GFP}(\%)=\frac{Y_{\text{max}}\text{time}}{K_{m}+\text{time}}$$

where Nuclear GFP (%) is the fraction of nuclei containing GFP (i.e., CreA), *Y*_max_ is assumed to be 100%, and *K_m_* is the saturation constant. Values for *K_m_* for the wild-type control (CreA::GFP) and CreA phosphonull mutant (CreA^S319A^::GFP) are 17.8 and 4.2 s, respectively, implying that the phosphonull CreA takes approximately 4 times longer to enter the nucleus, and thus, the CreA^S319^ phosphosite appears important for efficient CreA nuclear import. However, the mutation does not completely prevent CreA from travelling to the nucleus. Previous work has shown that the deletion of the conserved, repressor, and alanine-rich subdomains affect CreA localization (28). The phosphosite investigated here, S319, is located very close to the conserved domain and is also proximal to the repressing domain. In one study, the deletion of the conserved subdomain abolished growth on cellulose, which was not observed for CreA^S319A^ (28). In addition, growth in glucose or cellulose was not affected by deletion of the repressor domain (28). Although the mechanism of CreA nuclear import remains incompletely understood, our results suggest that S319 phosphorylation regulates the dynamics of CreA nuclear transport.

10.1128/mBio.02825-18.2 FIG S2 Effects of CreA mutation (S319A) on different known CreA targets. Download FIG S2, JPG file, 0.1 MB. Copyright © 2019 Ribeiro et al. 2019 Ribeiro et al. This content is distributed under the terms of the Creative Commons Attribution 4.0 International license.

To determine whether expression levels of GFP-tagged, phosphonull CreA^S319A^ were similar to those of GFP-tagged CreA we grew fungi in minimal medium with glucose, isolated cellular proteins, and performed Western blotting (Fig. S3). We find a significant increase in expression of GFP-tagged phosphonull CreA^S319A^ but similar stabilities of CreA in both strains. While it is not clear why there was an increase in expression, both strains showed similar cellulase activity (Fig. 4B), implying that this increase did not impact cellular phenotype.

10.1128/mBio.02825-18.3 FIG S3 Western blot of CreA(S319A)::GFP compared to CreA::GFP. Download FIG S3, JPG file, 0.1 MB. Copyright © 2019 Ribeiro et al. 2019 Ribeiro et al. This content is distributed under the terms of the Creative Commons Attribution 4.0 International license.

### Conclusion.

In this work, we were able to comprehensively identify both direct and indirect targets of PKA phosphorylation in A. nidulans, as well as genes whose expression depends on the presence of PKA. In the PKA deletion strain, transcription factor CreA was found to be down phosphorylated at S319. Recently, we have immunoprecipitated A. nidulans CreA:TAP under repressing and derepressing conditions using glucose or xylan, respectively, as the single carbon source (47). In both conditions, we have not seen PkaA interacting physically with CreA (47). Together with the results shown here, these data strongly indicate that PkaA is not physically interacting with CreA or that this interaction is very transient and/or weak. Thus, it is quite likely that CreA S319 is phosphorylated by another kinase regulated by PkaA and thus is an indirect target of PKA. Functional analysis of CreA^S319^ revealed its importance for timely nuclear import. Besides providing a broad overview of the PKA regulatory network in a model filamentous fungus, our results will also facilitate detailed functional investigation of PKA phosphorylation events and their role(s) in growth and development.

## MATERIALS AND METHODS

### Strains, cell culture, and media composition.

Two strains were used for phosphoproteomic and transcriptomic analysis, strains FGSC A1405 (also described as WT in this work) and FGSC A1358 (PKA deletion mutant). The *PkaA*::*GFP* construction used in this work was made by the method of de Assis et al. (29). Strains AGB551 (Δ*nkuA*::*argB pyrG89 pyroA4 veA*+) and TNO2A3 (*pyroA4 pyrG89 chaA1 ΔnKuA*::*argB*) were used as a genetic background. *CreA*::*GFP* and *CreA*(*S319A*)::*GFP* constructions were made by the method of de Assis et al. (29). TNO2A3 was used as a genetic background. The two media used were YGV (2% glucose, 0.5% yeast extract, and vitamins) and minimal medium. Minimal medium consists of 1% (wt/vol) carbon source (50 ml of salt solution [120 g/liter NaNO_3_, 10.4 g/liter KCl, 30 g/liter KH_2_PO_4_, and 10.4 g/liter MgSO_4_]), 1 ml of 5× trace elements (22.0 g/liter ZnSO_4_, 11 g/liter boric acid, 5 g/liter MnCl_2_, 5 g/liter FeSO_4_, 1.6 g/liter CoCl, 1.6 g/liter CuSO_4_, 1.1 g/liter (NH_4_)_2_MoO_4_, and 50 g/liter EDTA), and 0.01 g/liter pyridoxine. Brieﬂy, 10^7^ fresh spores were inoculated into 50 ml of liquid YGV (pH 3.5) and grown at 28°C and 250 rpm for 13 h. This culture, comprised of germinating spores, was poured into 2.8-liter baffled Fernbach flasks holding 1.2 liters of YGV (pH 6.5) and incubated at 28°C and 250 rpm for 20 h.

### Extraction and digestion of intracellular proteins for phosphoproteomics.

Mycelia from FGSC A1405 (WT) and FGSC A1358 (PKA deletion) were harvested after 20 h of growth (during logarithmic growth phase; growth curves shown in Fig. S4 in the supplemental material) by passing the culture through a Buchner funnel and immediately freezing mycelia in liquid nitrogen. Frozen mycelia were ground into powder by using a mortar and pestle, and RIPA buffer (Thermo Fisher Scientific) was used to extract protein with a 15-min incubation at 4°C and constant agitation. The mixture was centrifuged at 10,000 × *g* for 20 min, and the supernatant was collected and processed for protein concentration by BCA protein assay (ThermoFisher Scientific Inc., Rockford, IL). Five hundred micrograms of protein was purified by 3-kDa-molecular-weight cutoff ultrafiltration (Centriprep YM-3; Millipore, Billerica, MA) followed by trichloroacetic acid (TCA) precipitation. TCA was added to protein extract at 10% final concentration (wt/vol, made fresh), vortexed for 15 s, and placed on ice for a minimum of 20 min. The samples were centrifuged at 14,000 × *g* and 4°C for 15 min, and the supernatant was removed. The pellet was washed with cold acetone and then centrifuged at 14,000 × *g* and 4°C for 10 min. The acetone was discarded, and the samples were air dried. Protein (500 μg) was reduced with Tris(2-carboxyethyl)phosphine, alkylated with iodoacetamide, and digested with sequencing-grade trypsin (Promega, Madison, WI). Peptides were lyophilized, and phosphopeptides were enriched using Pierce TiO2 Phosphopeptide Enrichment and Clean-up kit (Thermo Fisher Scientific) according to the manufacturer’s instructions. Samples were then lyophilized.

10.1128/mBio.02825-18.4 FIG S4 Growth curves of the WT and PKA knockout strains. Download FIG S4, JPG file, 0.1 MB. Copyright © 2019 Ribeiro et al. 2019 Ribeiro et al. This content is distributed under the terms of the Creative Commons Attribution 4.0 International license.

### LC-MS/MS for phosphopeptides.

After phospho-enrichment, samples were resolved in 10 μl of acetonitrile (ACN)/H_2_O/fatty acid (FA) (5/95/0.1, vol/vol/vol) for LC-MS/MS analysis. The samples were analyzed in triplicate on a nanoLC (NanoAcquity; Waters Corporation, Milford, MA) coupled to a Orbitrap Fusion Tribrid mass spectrometer (Thermo Fisher, San Jose, CA). For each run, 2 μl of sample was loaded on a C_18_ precolumn (100-μm inner diameter [i.d.] by 20 mm; 5 μm) at a flow rate of 4 μl/min for 5 min, using a loading buffer of ACN/H_2_O/FA (5/95/0.1, vol/vol/vol). Peptide separation was performed on a C_18_ analytical column (75-μm i.d. by 180 mm; 5 μm) at a flow rate of 250 nl/min in a 95-min gradient by using mobile phase A (0.1% formic acid in water) and mobile phase B (0.1% FA in acetonitrile). The gradient elution started at 5% mobile phase B, increased to 35% at 60 min and then 80% at 65 min, and was held at 80% for 5 min before a 25-min reequilibration at 5%. The eluting peptides were interrogated with an Orbitrap Fusion mass spectrometer running a data-dependent LC-MS/MS method with the Top Speed decisions selection. FTMS1 spectra were collected using the following parameters: scan range of 350 to 1,800 *m/z*, resolving power of 120K, AGC target 4E5, and maximum injection time of 50 ms. ITMS2 spectra were collected using the following parameters: rapid scan rate, CID NCE 35, 1.6 *m/z* isolation window, AGC target 1E4, and maximum injection time of 50 ms. MS2 precursors were selected for a 3-s cycle. Precursors with an assigned monoisotopic *m/z* and a charge state of 2 to 7 were interrogated. Precursors were filtered using a 60-s dynamic exclusion window.

### Data analysis for phosphoproteomics.

Raw mass spectrometric data were analyzed in the MaxQuant environment (50) v.1.5.0.0 and employed Andromeda for database search (51). The MS/MS spectra were matched against the Aspergillus nidulans Uniprot FASTA database. Enzyme specificity was set to trypsin, and the search included cysteine carbamidomethylation as a fixed modification and N-acetylation of protein, oxidation of methionine, and/or phosphorylation of Ser, Thr, and Tyr residues (STY) as variable modifications. Up to two missed cleavages were allowed for protease digestion, and peptides had to be fully tryptic. The maximum peptide and site false-discovery rates were specified as 0.01. We used Student test for testing differences between the means of two conditions. Two-tailed hypothesis test at the 0.05 level of significance was applied.

### RNA-seq analysis.

Samples for transcriptomics were obtained from the same cultures used for phosphoproteomic samples. Harvested cells were immediately frozen, crushed, and prepped for RNA-seq analysis. For RNA-seq analysis, sequencing libraries were constructed from RNA samples using TruSeq RNA Sample Preparation kit v2 (Illumina, Inc., San Diego, CA), and 100-bp reads were generated on the Illumina Genome Analyzer IIx platform (Illumina, Inc., San Diego, CA). RNA-seq output data were checked for base quality and aligned to the reference genome of Aspergillus nidulans (www.aspgd.org/) using bowtie2, and the read counts were normalized using HT-Seq. Differential expression analysis was done using DESeq software (52). Genes were called differentially expressed based on log_2_ fold change, and significance is estimated for *P* values less than 0.01.

### Protein extraction for *pka*::*GFP* pulldown.

Crude protein extracts from mycelia were obtained by extraction from ground mycelia with B250 buffer (250 mM NaCl, 100 mM Tris-HCl [pH 7.5], 10% glycerol, 1 mM EDTA, and 0.1% NP-40) supplemented with 1.5 ml/liter 1 M DTT, 2 tablets/100 ml Complete-mini Protease Inhibitor Cocktail EDTA-free (Roche), 3 ml/liter 0.5 M benzamidine, 10 ml/liter 100× phosphatase inhibitors (10 M NaF, 5 M Na vanadate, 8 M β-glycerol phosphate), and 10 ml/liter 100 mM PMSF.

### GFP tag protein purification.

The GFP-tagged protein complex was purified from crude protein extracts incubating 4 g ground mycelia in 4 ml B250 buffer described above. The total protein lysate was incubated with 40 μl GFP-Trap magnetic beads (Chromotek) for 4 h at 4°C. Beads were harvested using magnetics hack and washed three times using B250 buffer without DTT, being added only in the last wash step. The precipitated complex was digested using sequencing-grade modified trypsin (catalog no. V5117; Promega), and the desalinization step was made using Zip-Tip (catalog no. ZTC18S096; Millipore) according to the manufacturer’s instructions.

### LC-MS/MS protein identification for *pka*::*GFP* pulldown.

Digested peptides were separated using reversed-phase liquid chromatography with an RSLCnano Ultimate 3000 system (Thermo Scientific) followed by mass identification with an Orbitrap Velos Pro mass spectrometer (Thermo Scientific). Chromatographically separated peptides were on-line ionized by nanoelectrospray ionization (nESI) using the Nanospray Flex Ion Source (Thermo Scientific) at 2.4 kV and continuously transferred into the mass spectrometer. Full scans within *m/z* of 300 to 1,850 were recorded by the Orbitrap-FT analyzer at a resolution of 30.000 (using *m/z* 445.120025 as lock mass) with parallel data-dependent top 10 MS2- fragmentation in the LTQ Velos Pro linear ion trap. LC-MS method programming and data acquisition were performed with the software XCalibur 2.2 (Thermo Scientific) and method/raw data validation with the program RawMeat 2.1 (Vast Scientific).

MS/MS2 data processing for protein analysis and identification was done with either MaxQuant quantitative proteomic software in conjunction with Perseus software for statistical analysis or the Proteome Discoverer 1.3 (PD) (Thermo Scientific) and the Discoverer Daemon 1.3 (Thermo Scientific) software using the Sequest (and/or Mascot) peptide analysis algorithm(s) and organism-specific taxon-defined protein databases extended by the most common contaminants.

The interactions identified were submitted to filtering subtracting unspecific interactions from AGB551 as the genetic background strain grown under the same experimental conditions. MS interactions from duplicates for each time point with at least two unique peptides identified per protein were applied during the analysis; PkaA was identified in all pulldown assays as an internal control for tagged protein precipitation.

### GFP-Trap immunoprecipitation.

Total protein extracts from mycelia were obtained by extraction from ground mycelia with B250 extraction buffer (250 mM NaCl, 100 mM Tris-HCl [pH 7.5], 10% glycerol, 1 mM EDTA, and 0.1% NP-40) supplemented with 1.5 ml/liter of 1 M DTT, 1 pill/10 ml of the Complete-mini Protease Inhibitor Cocktail EDTA-free (Roche), 3 ml/liter of 0.5 M benzamidine, 100 μl/10 ml of phosphatase inhibitors P0044 (Sigma), and 10 ml/liter of 100 mM PMSF. GFP-Trap beads (40 μl) were equilibrated with 500 μl of extraction buffer and then centrifuged at 3,000 rpm at 4°C for 1 min; all the next wash steps were done under the same conditions. Five milligrams of total protein extract was incubated with GFP-Trap beads for 4 h at 4°C in a rotator shaker; after the incubation, the beads were washed two times using extraction buffer without DTT and one more additional wash with DTT. The supernatant was removed, and the GFP-Trap beads were incubated with 40 μl of 1× SDS sample buffer and boiled at 95°C for 5 min. The proteins (total) released from the beads were loaded into SDS-polyacrylamide gel (12%) for Western blotting as previously described (47).

### Fluorescence microscopy for nuclear localization.

For microscopy experiments, spores were grown on coverslips for image analysis. Briefly, coverslips bearing freshly harvested spores were inoculated in a small petri dish filled with 3 ml minimal medium supplemented with 1% Avicel or 1% glucose for 16 h at 22°C. When mycelia were grown in minimal medium supplemented with 1% Avicel or 1% ethanol, 1% glucose was added after 16 h. Images were taken after 2.5, 5, 10, 15, 30, 60, 80, and 100 min. Mycelia mounted on coverslips were washed with phosphate-buffered saline (PBS) (140 mM NaCl, 2 mM KCl, 10 mM NaHPO_4_, 1.8 mM KH_2_PO_4_ [pH 7.4]). The mycelia were then stained with 100 ng/ml Hoechst 33258 (Molecular Probes) for 2 min. The mycelia were washed again in water and examined using a Zeiss epifluorescence microscope with excitation wavelengths of 359 and 498 nm and emission wavelengths of 461 and 516 nm for Hoechst and GFP, respectively. Phase-contrast bright-field and fluorescent images were captured with an AxioCam camera (Carl Zeiss) and processed using AxioVision software version 3.1. Representative images (Fig. S1) show nuclear localization. Brightness and contrast have been altered identically in all images to better show CreA::GFP localization. To assess the rate of nuclear localization, data were subjected to nonlinear regression (equation 1) using PolyMath software (CACHE Corp.) to determine values of *K_m_*.

### Cellulolytic activity.

Enzymatic activity was measured by colorimetric assay using Avicel as a substrate. The reducing sugars were determined by the Miller procedure using glucose as the control (53). The reaction mixture (0.05 ml substrate [1%, wt/vol] in 50 mM sodium acetate buffer [pH 5.0] and 0.05 ml enzyme solution) was incubated at 50°C for 30 min. The reaction was stopped by adding 0.1 ml of dinitrosalicylic acid (DNS) and immediately boiling for 5 min. Quantification of the reducing sugars released as a result of enzyme activity was estimated by *A*_540_ measurements, where one unit of enzymatic activity was defined as the amount of enzyme that produced 1 μmol min^−1^ of reducing sugars.

### Site-directed mutagenesis of CreA.

The substitution of CreA S319 with alanine was introduced by site-directed mutagenesis, using oligonucleotides containing the mutation. The DNA cassette was constructed containing 1 kb of the upstream *creA* region, *creA* mutated, *pyrG*, and 1 kb of the downstream *creA* region. The plasmid pCreA/GFP containing the whole cassette was built using plasmid pUC19 as a backbone via Gibson assembly (54). The mutated CreA (S319A) was constructed by inverse PCR (55) of the pCreA/GFP plasmid, generating the construct pCreAS319A/GFP containing the mutated CreA gene. After fully sequencing pCreAS319A/GFP, the CreAS319A/GFP/pyrG cassette was PCR amplified and used to transform A. nidulans TNO2A3. Genomic DNA extraction was conducted for six transformants and submitted for sequencing to verify the absence of any additional base changes introduced into *creA* as a result of transformation.

### Data availability.

The RNA-seq data from this publication have been deposited into the GEO database (https://www.ncbi.nlm.nih.gov/geo/) and assigned the identifier or accession no. GSE116579.

## References

[1] Landry CR, Levy ED, Abd Rabbo D, Tarassov K, Michnick SW 2013 XExtracting insight from noisy cellular networks. Cell 155:983–989. doi:10.1016/j.cell.2013.11.003. 24267884

[2] Vidal M, Cusick ME, Barabási A-L 2011 Interactome networks and human disease. Cell 144:986–998. doi:10.1016/j.cell.2011.02.016. 21414488 PMC3102045

[3] Holsbeeks I, Lagatie O, Popova Y, Rolland F, Stolz F, Van De Velde S, Van Dijck P, Vandormael P, Van Nuland A, Van Roey K, Van Zeebroeck G, Yan B 2005 Nutrient sensing systems for rapid activation of the protein kinase A pathway in yeast. Biochem Soc Trans 33:253–256. doi:10.1042/BST0330253. 15667319

[4] Casado C, González A, Platara M, Ruiz A, Ariño J 2011 The role of the protein kinase A pathway in the response to alkaline pH stress in yeast. Biochem J 438:523–533. doi:10.1042/BJ20110607. 21749328 PMC3253439

[5] Freitas FZ, de Paula RM, Barbosa LCB, Terenzi HF, Bertolini MC 2010 cAMP signaling pathway controls glycogen metabolism in Neurospora crassa by regulating the glycogen synthase gene expression and phosphorylation. Fungal Genet Biol 47:43–52. doi:10.1016/j.fgb.2009.10.011. 19883780

[6] Fuller KK, Richie DL, Feng X, Krishnan K, Stephens TJ, Wikenheiser-Brokamp KA, Askew DS, Rhodes JC 2011 Divergent protein kinase A isoforms co-ordinately regulate conidial germination, carbohydrate metabolism and virulence in Aspergillus fumigatus. Mol Microbiol 79:1045–1062. doi:10.1111/j.1365-2958.2010.07509.x. 21210869 PMC3065306

[7] Liebmann B, Müller M, Braun A, Brakhage AA 2004 The cyclic AMP-dependent protein kinase A network regulates development and virulence in Aspergillus fumigatus. Infect Immun 72:5193–5203. doi:10.1128/IAI.72.9.5193-5203.2004. 15322014 PMC517480

[8] Søberg K, Moen LV, Skålhegg BS, Laerdahl JK 2017 Evolution of the cAMP-dependent protein kinase (PKA) catalytic subunit isoforms. PLoS One 12:e0181091. doi:10.1371/journal.pone.0181091. 28742821 PMC5526564

[9] Santangelo GM 2006 Glucose signaling in Saccharomyces cerevisiae. Microbiol Mol Biol Rev 70:253–282. doi:10.1128/MMBR.70.1.253-282.2006. 16524925 PMC1393250

[10] Ribeiro LFC, Chelius CL, Harris SD, Marten MR 2017 Insights regarding fungal phosphoproteomic analysis. Fungal Genet Biol 104:38–44. doi:10.1016/j.fgb.2017.03.003. 28288883

[11] Oliver BG, Panepinto JC, Fortwendel JR, Smith DL, Askew DS, Rhodes JC 2002 Cloning and expression of pkaC and pkaR, the genes encoding the cAMP-dependent protein kinase of Aspergillus fumigatus. Mycopathologia 154:85–91. doi:10.1023/A:1015533406565. 12086105

[12] Brown NA, Ries LNA, Goldman GH 2014 How nutritional status signalling coordinates metabolism and lignocellulolytic enzyme secretion. Fungal Genet Biol 72:48–63. doi:10.1016/j.fgb.2014.06.012. 25011009

[13] Ziv C, Gorovits R, Yarden O 2008 Carbon source affects PKA-dependent polarity of Neurospora crassa in a CRE-1-dependent and independent manner. Fungal Genet Biol 45:103–116. doi:10.1016/j.fgb.2007.05.005. 17625933

[14] Huberman LB, Liu J, Qin L, Glass NL 2016 Regulation of the lignocellulolytic response in filamentous fungi. Fungal Biol Rev 30:101–111. doi:10.1016/j.fbr.2016.06.001.

[15] Wang Y, Pierce M, Schneper L, Güldal CG, Zhang X, Tavazoie S, Broach JR 2004 Ras and Gpa2 mediate one branch of a redundant glucose signaling pathway in yeast. PLoS Biol 2:E128. doi:10.1371/journal.pbio.0020128. 15138498 PMC406390

[16] Moir RD, Lee J, Haeusler RA, Desai N, Engelke DR, Willis IM 2006 Protein kinase A regulates RNA polymerase III transcription through the nuclear localization of Maf1. Proc Natl Acad Sci U S A 103:15044–15049. doi:10.1073/pnas.0607129103. 17005718 PMC1622776

[17] Liu X, Li H, Liu Q, Niu Y, Hu Q, Deng H, Cha J, Wang Y, Liu Y, He Q 2015 Role for protein kinase A in the Neurospora circadian clock by regulating White Collar-independent *frequency* transcription through phosphorylation of RCM-1. Mol Cell Biol 35:2088–2102. doi:10.1128/MCB.00709-14. 25848091 PMC4438235

[18] Gorner W, Durchschlag E, Martinez-Pastor MT, Estruch F, Ammerer G, Hamilton B, Ruis H, Schuller C 1998 Nuclear localization of the C2H2 zinc finger protein Msn2p is regulated by stress and protein kinase A activity. Genes Dev 12:586–597. doi:10.1101/gad.12.4.586. 9472026 PMC316529

[19] Wei M, Fabrizio P, Hu J, Ge H, Cheng C, Li L, Longo VD 2008 Life span extension by calorie restriction depends on Rim15 and transcription factors downstream of Ras/PKA, Tor, and Sch9. PLoS Genet 4:e13. doi:10.1371/journal.pgen.0040013. 18225956 PMC2213705

[20] Klein C, Struhl K 1994 Protein kinase A mediates growth-regulated expression of yeast ribosomal protein genes by modulating RAP1 transcriptional activity. Mol Cell Biol 14:1920–1928. doi:10.1128/MCB.14.3.1920. 8114723 PMC358550

[21] Cytryńska MI, Frajnt M, Jakubowicz T 2001 Saccharomyces cerevisiae pyruvate kinase Pyk1 is PKA phosphorylation substrate in vitro. FEMS Microbiol Lett 203:223–227. doi:10.1111/j.1574-6968.2001.tb10845.x. 11583852

[22] Barrett L, Orlova M, Maziarz M, Kuchin S 2012 Protein kinase A contributes to the negative control of SNF1 protein kinase in Saccharomyces cerevisiae. Eukaryot Cell 11:119–128. doi:10.1128/EC.05061-11. 22140226 PMC3272905

[23] Hedbacker K, Townley R, Carlson M 2004 Cyclic AMP-dependent protein kinase regulates the subcellular localization of Snf1-Sip1 protein kinase. Mol Cell Biol 24:1836–1843. doi:10.1128/MCB.24.5.1836-1843.2004. 14966266 PMC350547

[24] Cherry JR, Johnson TR, Dollard C, Shuster JR, Denis CL 1989 Cyclic AMP-dependent protein kinase phosphorylates and inactivates the yeast transcriptional activator ADR1. Cell 56:409–419. doi:10.1016/0092-8674(89)90244-4. 2644045

[25] Lee J, Moir RD, Willis IM 2015 Differential phosphorylation of RNA polymerase III and the initiation factor TFIIIB in Saccharomyces cerevisiae. PLoS One 10:e0127225. doi:10.1371/journal.pone.0127225. 25970584 PMC4430316

[26] Budovskaya YV, Stephan JS, Deminoff SJ, Herman PK 2005 An evolutionary proteomics approach identifies substrates of the cAMP-dependent protein kinase. Proc Natl Acad Sci U S A 102:13933–13938. doi:10.1073/pnas.0501046102. 16172400 PMC1236527

[27] Alam MA, Kelly JM 2017 Proteins interacting with CreA and CreB in the carbon catabolite repression network in Aspergillus nidulans. Curr Genet 63:669–683. doi:10.1007/s00294-016-0667-2. 27915380

[28] Ries LNA, Beattie SR, Espeso EA, Cramer RA, Goldman GH 2016 Diverse regulation of the CreA carbon catabolite repressor in Aspergillus nidulans. Genetics 203:335–352. doi:10.1534/genetics.116.187872. 27017621 PMC4858783

[29] de Assis LJ, Ries LNA, Savoldi M, Dos Reis TF, Brown NA, Goldman GH 2015 Aspergillus nidulans protein kinase A plays an important role in cellulase production. Biotechnol Biofuels 8:213. doi:10.1186/s13068-015-0401-1. 26690721 PMC4683954

[30] Cziferszky A, Mach RL, Kubicek CP 2002 Phosphorylation positively regulates DNA binding of the carbon catabolite repressor Cre1 of Hypocrea jecorina (Trichoderma reesei). J Biol Chem 277:14688–14694. doi:10.1074/jbc.M200744200. 11850429

[31] Smith FC, Davies SP, Wilson WA, Carling D, Hardie DG 1999 The SNF1 kinase complex from Saccharomyces cerevisiae phosphorylates the transcriptional repressor protein Mig1p in vitro at four sites within or near regulatory domain 1. FEBS Lett 453:219–223. doi:10.1016/S0014-5793(99)00725-5. 10403407

[32] De Vit MJ, Waddle JA, Johnston M 1997 Regulated nuclear translocation of the Mig1 glucose repressor. Mol Biol Cell 8:1603–1618. doi:10.1091/mbc.8.8.1603. 9285828 PMC276179

[33] Brown NA, De Gouvea PF, Krohn NG, Savoldi M, Goldman GH 2013 Functional characterisation of the non-essential protein kinases and phosphatases regulating Aspergillus nidulans hydrolytic enzyme production. Biotechnol Biofuels 6:91. doi:10.1186/1754-6834-6-91. 23800192 PMC3698209

[34] De Souza CP, Hashmi SB, Osmani AH, Andrews P, Ringelberg CS, Dunlap JC, Osmani SA 2013 Functional analysis of the Aspergillus nidulans kinome. PLoS One 8:e58008. doi:10.1371/journal.pone.0058008. 23505451 PMC3591445

[35] Franck WL, Gokce E, Randall SM, Oh Y, Eyre A, Muddiman DC, Dean RA 2015 Phosphoproteome analysis links protein phosphorylation to cellular remodeling and metabolic adaptation during Magnaporthe oryzae appressorium development. J Proteome Res 14:2408–2424. doi:10.1021/pr501064q. 25926025 PMC4838196

[36] Leonhardt Y, Kakoschke SC, Wagener J, Ebel F 2017 Lah is a transmembrane protein and requires Spa10 for stable positioning of Woronin bodies at the septal pore of Aspergillus fumigatus. Sci Rep 7:44179. doi:10.1038/srep44179. 28281662 PMC5345055

[37] Schwartz D, Chou MF, Church GM 2009 Predicting protein post-translational modifications using meta-analysis of proteome scale data sets. Mol Cell Proteomics 8:365–379. doi:10.1074/mcp.M800332-MCP200. 18974045 PMC2634583

[38] Lv DW, Li X, Zhang M, Gu AQ, Zhen SM, Wang C, Li XH, Yan YM 2014 Large-scale phosphoproteome analysis in seedling leaves of Brachypodium distachyon L. BMC Genomics 15:375. doi:10.1186/1471-2164-15-375. 24885693 PMC4079959

[39] Kanzaki M 2006 Insulin receptor signals regulating GLUT4 translocation and actin dynamics. Endocr J 53:267–293. doi:10.1507/endocrj.KR-65. 16702775

[40] Garai Á, Zeke A, Gógl G, Töro I, Fördos F, Blankenburg H, Bárkai T, Varga J, Alexa A, Emig D, Albrecht M, Reményi A 2012 Specificity of linear motifs that bind to a common mitogen-activated protein kinase docking groove. Sci Signal 5:ra74. doi:10.1126/scisignal.2003004. 23047924 PMC3500698

[41] David H, Özçelik IŞ, Hofmann G, Nielsen J 2008 Analysis of Aspergillus nidulans metabolism at the genome-scale. BMC Genomics 9:163. doi:10.1186/1471-2164-9-163. 18405346 PMC2386489

[42] Bernardo SMH, Gray KA, Todd RB, Cheetham BF, Katz ME 2007 Characterization of regulatory non-catalytic hexokinases in Aspergillus nidulans. Mol Genet Genomics 277:519–532. doi:10.1007/s00438-006-0203-z. 17226029

[43] Cao C, Wu M, Bing J, Tao L, Ding X, Liu X, Huang G 2017 Global regulatory roles of the cAMP/PKA pathway revealed by phenotypic, transcriptomic and phosphoproteomic analyses in a null mutant of the PKA catalytic subunit in Candida albicans. Mol Microbiol 105:46–64. doi:10.1111/mmi.13681. 28370450

[44] O’Meara TR, Xu W, Selvig KM, O’Meara MJ, Mitchell AP, Alspaugh JA 2014 The Cryptococcus neoformans Rim101 transcription factor directly regulates genes required for adaptation to the host. Mol Cell Biol 34:673–684. doi:10.1128/MCB.01359-13. 24324006 PMC3911494

[45] Ries LNA, Beattie S, Cramer RA, Goldman GH 2018 Overview of carbon and nitrogen catabolite metabolism in the virulence of human pathogenic fungi. Mol Microbiol 107:277–297. doi:10.1111/mmi.13887. 29197127 PMC5777862

[46] Sciuto MR, Warnken U, Schnölzer M, Valvo C, Brunetto L, Boe A, Biffoni M, Krammer PH, De Maria R, Haas TL 2018 Two-step coimmunoprecipitation (TIP) enables efficient and highly selective isolation of native protein complexes. Mol Cell Proteomics 17:993–1009. doi:10.1074/mcp.O116.065920. 29217617 PMC5930409

[47] de Assis LJ, Ulas M, Ries LNA, El Ramli NAM, Sarikaya-Bayram O, Braus GH, Bayram O, Goldman GH 2018 Regulation of Aspergillus nidulans CreA-mediated catabolite repression by the F-box proteins Fbx23 and Fbx47. mBio 9:e00840-18. doi:10.1128/mBio.00840-18. 29921666 PMC6016232

[48] Roy P, Lockington RA, Kelly JM 2008 CreA-mediated repression in Aspergillus nidulans does not require transcriptional auto-regulation, regulated intracellular localisation or degradation of CreA. Fungal Genet Biol 45:657–670. doi:10.1016/j.fgb.2007.10.016. 18063396

[49] Alam MA, Kamlangdee N, Kelly JM 2017 The CreB deubiquitinating enzyme does not directly target the CreA repressor protein in Aspergillus nidulans. Curr Genet 63:647–667. doi:10.1007/s00294-016-0666-3. 27878624

[50] Cox J, Mann M 2008 MaxQuant enables high peptide identification rates, individualized p.p.b.-range mass accuracies and proteome-wide protein quantification. Nat Biotechnol 26:1367–1372. doi:10.1038/nbt.1511. 19029910

[51] Cox J, Neuhauser N, Michalski A, Scheltema RA, Olsen JV, Mann M 2011 Andromeda: a peptide search engine integrated into the MaxQuant environment. J Proteome Res 10:1794–1805. doi:10.1021/pr101065j. 21254760

[52] Anders S, Huber W 2010 Differential expression analysis for sequence count data. Genome Biol 11:R106. doi:10.1186/gb-2010-11-10-r106. 20979621 PMC3218662

[53] Miller GL 1959 Use of dinitrosalicylic acid reagent for determination of reducing sugar. Anal Chem 31:426–428. doi:10.1021/ac60147a030.

[54] Gibson DG, Young L, Chuang RY, Venter JC, Hutchison CA, Smith HO 2009 Enzymatic assembly of DNA molecules up to several hundred kilobases. Nat Methods 6:343–345. doi:10.1038/nmeth.1318. 19363495

[55] Jain PC, Varadarajan R 2014 A rapid, efficient, and economical inverse polymerase chain reaction-based method for generating a site saturation mutant library. Anal Biochem 449:90–98. doi:10.1016/j.ab.2013.12.002. 24333246

