# Supplementary figures and images for: Comprehensive Analysis of Aspergillus nidulans PKA Phosphorylome Identifies a Novel Mode of CreA Regulation
Source: mBio. 2019 Apr 30;10(2):e02825-18. doi: 10.1128/mBio.02825-18 (PMC6495382; doi:10.1128/mBio.02825-18)

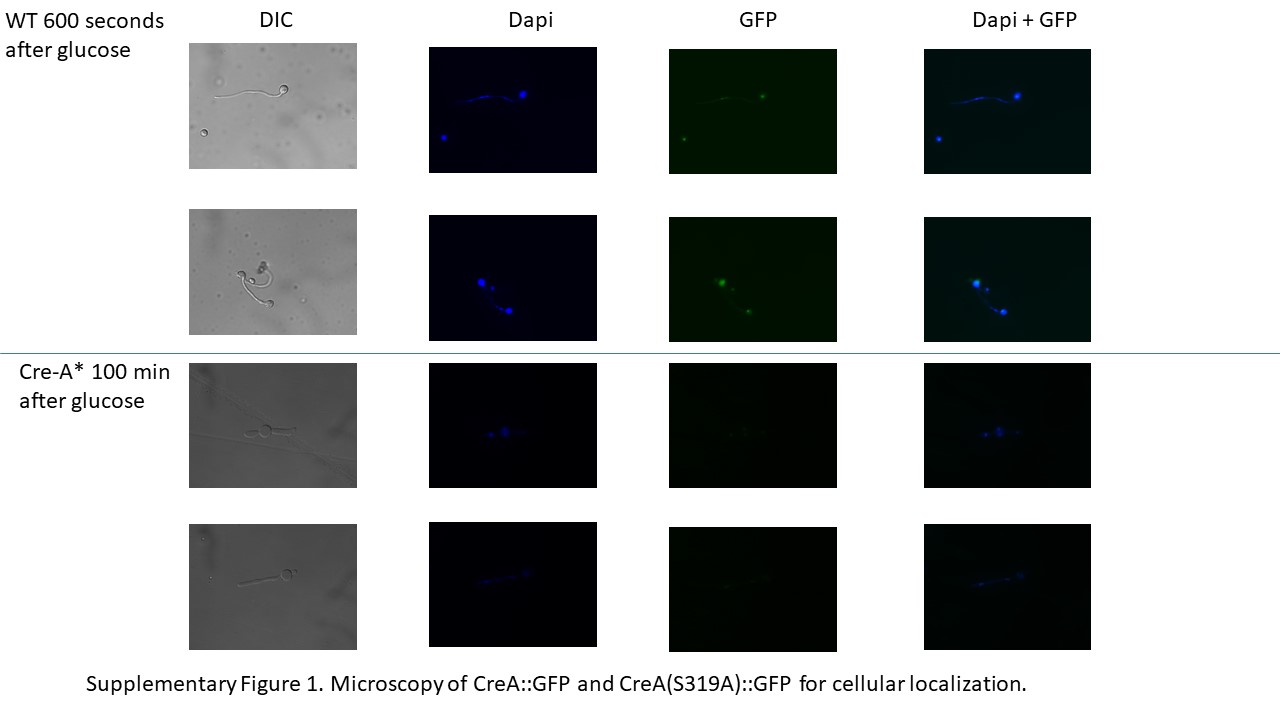

Supplement: FIG S1 [file mBio.02825-18-sf001.jpg]

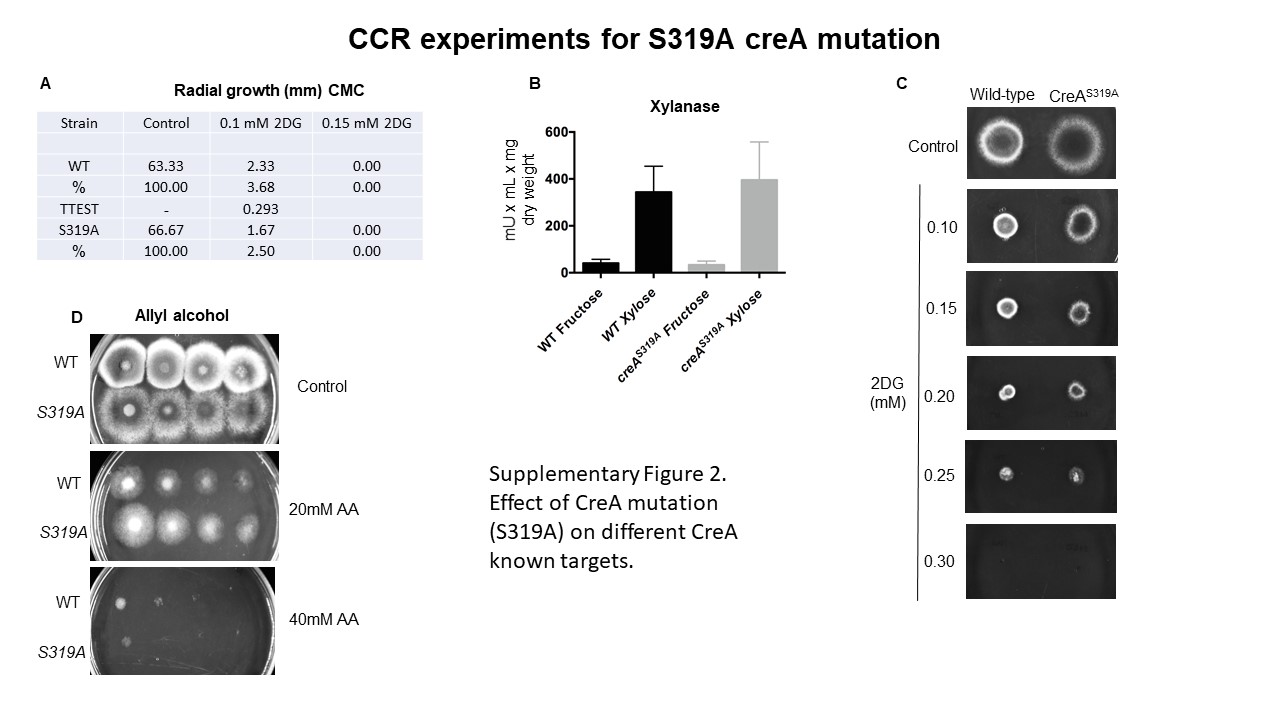

Supplement: FIG S2 [file mBio.02825-18-sf002.jpg]

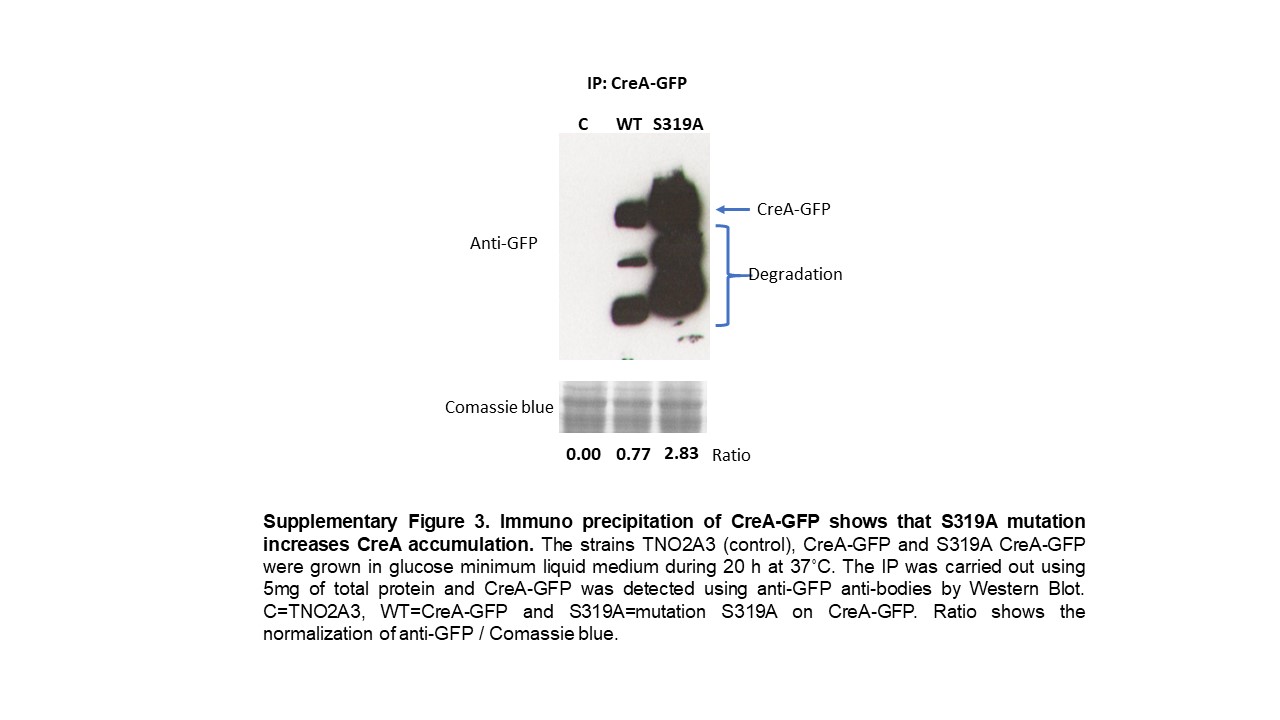

Supplement: FIG S3 [file mBio.02825-18-sf003.jpg]

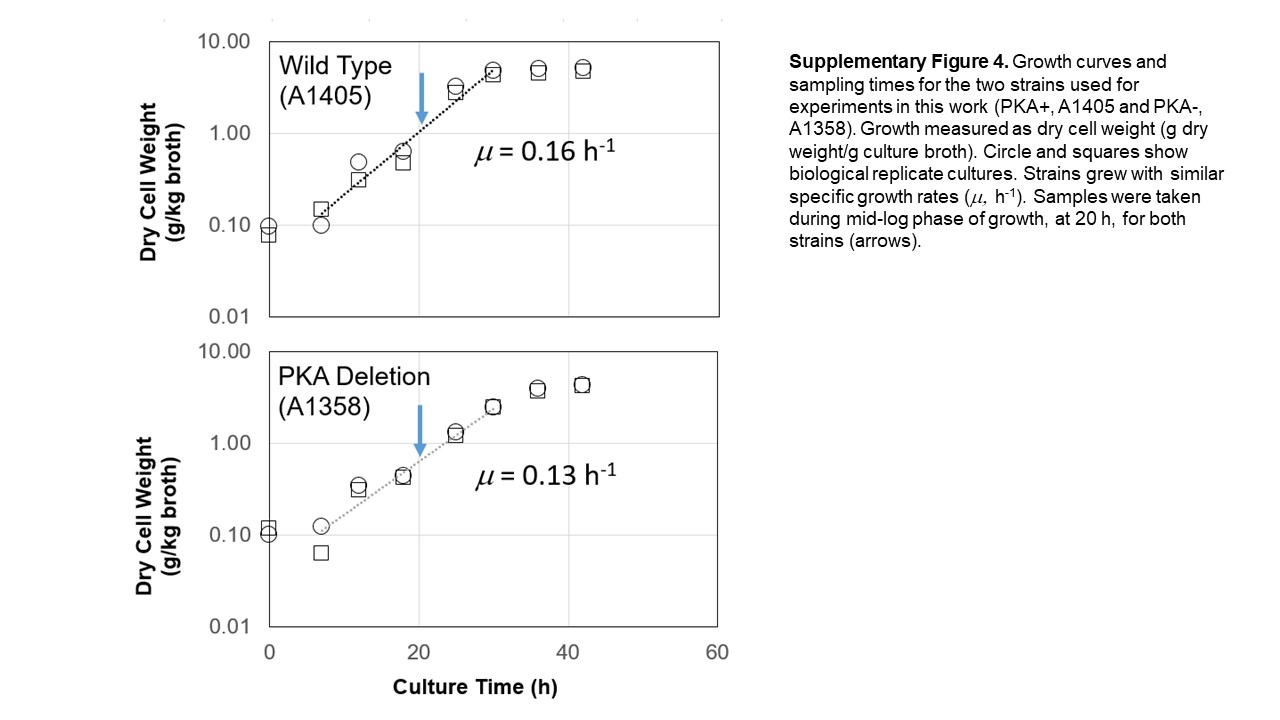

Supplement: FIG S4 [file mBio.02825-18-sf004.jpg]
